# Development of an Anti-Zearalenone Nanobody Phage Display Library and Preparation of Specific Nanobodies

**DOI:** 10.3390/cimb47030157

**Published:** 2025-02-27

**Authors:** Ying Zeng, Yiying Hu, Ganying Chen, Qingqing Feng, Ruiting Wang, Zhilin Zhang, Jinxian Chen, Junbin Liao, Danrong Lin, Wei Zhu

**Affiliations:** 1 School of Public Health, Southern Medical University, Guangzhou 510515, China; zying@i.smu.edu.cn (Y.Z.); 13580445856@163.com (Y.H.); 18923474095@163.com (J.L.); 2 Department of Scientific Research Management, Guangzhou Center for Disease Control and Prevention (Guangzhou Health Supervision Institute), Guangzhou 510440, China; cgy5802@163.com (G.C.); QQF1998@163.com (Q.F.); 18785071049@163.com (R.W.); zzl18athlom@gmail.com (Z.Z.); jx1319418878@163.com (J.C.); lindanrong927@163.com (D.L.)

**Keywords:** zearalenone, phage display, biopanning, prokaryotic expression, nanobody

## Abstract

Zearalenone (ZEN), a toxic estrogenic mycotoxin in cereals, threatens human and animal health through reproductive, immune, and cytotoxic effects, necessitating sensitive detection methods. While nanobodies offer advantages over conventional antibodies for on-site ZEN detection, their application remains unexplored. This study aimed to develop an anti-ZEN nanobody derived from an anti-ZEN phage display nanobody library. An alpaca was immunized with a ZEN-bovine serum albumin (ZEN-BSA) antigen, achieving peak serum antibody titers (1:25,600) following four immunizations. A high-capacity phage display nanobody library (1.0 × 10^11^ plaque-forming units/mL) was constructed. Following four rounds of biopanning, an enrichment factor of 479 was achieved. Phage ELISA screening identified six phage display nanobodies with specific ZEN-binding activity, and multiple sequence alignment revealed four unique nanobody sequences. The selected phage display nanobody, designated phage-V44, was expressed and purified, and its presence was validated by SDS-PAGE and western blotting, which detected a single approximately 17 kDa band consistent with the expected nanobody size. We established a working curve for an indirect competitive enzyme-linked immunoassay (ELISA) for ZEN, which showed an IC50 value of 7.55 ng/mL. The specificity and affinity of the V44 were also verified. Collectively, the study successfully constructed an anti-ZEN phage display nanobody library, screened four specific ZEN-binding phage display nanobodies, and prepared the anti-ZEN nanobody V44. Thereby establishing a foundation for the nanobody’s future integration into rapid on-site detection methods for ZEN in both animal feed and human food products.

## 1. Introduction

Zearalenone (ZEN), also known as F-2 toxin, a non-steroidal estrogenic mycotoxin first isolated from moldy maize by Stob et al. in 1962 [1], is produced by *Fusarium* species, such as *F. graminearum*, *F. semitectum*, *F. culmorum*, and *F. equiseti* [2]. Over fifteen ZEN metabolites have been identified, including α-Zearalenol (α-ZEL), β-Zearalenol (β-ZEL), α-Zearalanol (α-ZAL), β-Zearalanol (β-ZAL), and Zearalanone (ZAN) [3]. ZEN and its derivatives’ contamination are widespread in cereals (e.g., corn, wheat, rice, sorghum) and derived products [4], posing significant health risks due to its structural mimicry of estrogen. The estrogenic activity of ZEN and its modified forms are classified in the following order: α-ZEL > α-ZAL > ZEN ≈ ZAN ≈ β-ZAL > β-ZEL. By binding estrogen receptors, ZEN disrupts reproductive hormone balance and damages the reproductive system [5]. As a potential target organ for estrogen-related disorders [6], ZEN can also impact the function of the body’s immune organs, resulting in either immune stimulation or immunosuppression. This can lead to changes in immune cell viability, proliferation, and function. Furthermore, ZEN triggers oxidative stress, apoptosis in human embryonic stem cells [7], and metabolic disruptions in animal models (e.g., altered lipid/glucose homeostasis, gut microbiota imbalance) [8]. Its multifaceted toxicity—spanning reproductive [9], immunotoxic [6], genotoxic [10], and cytotoxic [11] effects—has prompted strict international regulations limiting ZEN levels in food and feed [12]. In 1993, the WHO’s International Agency for Research on Cancer (IARC) classified ZEN as a Group 3 carcinogen, denoting possible evidence to classify its carcinogenicity in humans.

However, ZEN contamination persists in edible grains [13,14], feed ingredients [15,16], pet food, and biological samples [17,18,19] for various reasons, raising significant public health concerns due to its potential adverse effects. To safeguard food safety and human health, there is an urgent need for highly sensitive, rapid, accurate, efficient, and stable analytical methods for on-site detection of trace amounts of ZEN. Current detection approaches fall into three categories: chemical, instrumental, and immunoassays. Chemical methods, such as thin-layer chromatography (TLC) [20], suffer from low sensitivity, high detection limits, and poor reproducibility. Instrumental techniques, like gas chromatography–mass spectrometry (GC–MS), coupled with immunoaffinity column purification and isotope internal standard (e.g., Luo et al. [21]), offer high sensitivity and specificity, with low detection limits and good reproducibility. However, their reliance on expensive equipment, complex sample pretreatment, and pure reference standards limits their utility for rapid on-site testing. In contrast, immunoassays [22] leverage antigen–antibody specificity and enable high sensitivity, selectivity, and adaptability to field applications. Therefore, the key to establishing and applying this detection method lies in high-quality antibodies. With the right antibodies, immunological analysis can provide a sensitive, rapid, and cost-effective solution for on-site detection of ZEN in food and feed, ensuring consumer safety and promoting public health.

Over the past few decades, significant progress has been made in producing antibodies against zearalenone (ZEN) using polyclonal and monoclonal techniques [23], which have been utilized across a spectrum of assays. Nonetheless, these antibodies encounter constraints due to low expression yields and structural instability. Camelid species have been identified as sources of a unique class of antibodies—heavy-chain antibodies (HCAbs)—that lack the light chain (LC) and the first constant domain (CH1), containing only the heavy chain [24]. The variable domain of these HCAbs, termed VHH or nanobodies (Nbs) [25], can be efficiently cloned using molecular biology techniques, demonstrating superior structural stability and binding affinity. With a molecular weight of approximately 15 kDa, nanobodies are the smallest known antigen-binding units, roughly one-tenth the size of conventional antibodies [26]. Nanobodies offer unique advantages, including excellent water solubility, stable physicochemical properties, enhanced tissue penetration, robust target specificity, suitability for large-scale production, and an increased capacity to mitigate the matrix effect [27]. While Nbs are increasingly used in biotoxin detection [28,29,30], their application in rapid on-site ZEN detection remains unreported, except for a study screening anti-idiotype Nbs from an alpaca naïve VHH phage display library as a ZEN hapten alternative [31]. Immune libraries, derived from antigen-exposed hosts, enable high-affinity Nb isolation due to in vivo affinity maturation, which drives B-lymphocyte proliferation and yields stable, high-affinity antibodies [32]. Consequently, this study aims to isolate highly stable and sensitive anti-ZEN Nbs from an immune phage display library, establishing a foundation for developing rapid on-site ZEN detection methods in animal feed and food products. Nanobodies hold transformative potential for improving food safety diagnostics, environmental monitoring, and clinical testing. Their integration into detection systems could enhance mycotoxin detection accuracy and efficiency. However, challenges such as scalable production, regulatory hurdles, and real-world validation must be addressed through interdisciplinary collaboration to translate this promise into public health solutions. Future research should prioritize these areas to enable the commercialization and broad adoption of Nb-based technologies.

## 2. Materials and Methods

### 2.1. Materials and Reagents

Unless otherwise indicated, all reagents used were of analytical grade. Zearalenone conjugated to bovine serum albumin (ZEN-BSA) and zearalenone conjugated to ovalbumin (ZEN-OVA) were sourced from Sangon Biotech (Shanghai, China). Freund’s complete and incomplete adjuvants, as well as Histopaque-1077, were procured from Sigma-Aldrich (St. Louis, MO, USA). Goat anti-Alpaca IgG heavy and light chain (HRP) conjugate was acquired from AlpVHHs Co., Ltd. (Chengdu, China). Trizol reagent was supplied by Invitrogen (Waltham, MA, USA). The PrimeScript II 1st Strand cDNA Synthesis Kit and TaKaRa Ex Taq DNA polymerase were products of Takara Bio (Dalian, China). The Gel Extraction Kit, Cycle Pure Kit, and Plasmid Mini Kit were all obtained from Omega Bio-Tek (Norcross, GA, USA). The restriction enzyme SfiI, Quick CIP, T4 DNA Ligase, and M13KO7 Helper Phage were purchased from New England Biolabs (Ipswich, MA, USA). The Anti-M13 Mouse Monoclonal Antibody (HRP) was a product of Yiqiao Shenzhou Technology Co., Ltd. (Beijing, China), while the HRP Anti-6× His tag antibody was sourced from Abcam (Cambridge, UK). Ni Sepharose Excel resin was provided by Cytiva (Marlborough, MA, USA). The pComb3XSS vector was a generous gift from Prof. Carlos F. Barbas’ laboratory at the Scripps Research Institute, RRID: Addgene_63890. Chemically competent *Escherichia coli* TG1 and TOP10F’ cells were both obtained from Weidi Biotechnology Co., Ltd. (Shanghai, China). Zearalenone liquid standard was procured from Pribolab Pte. Ltd. (Qingdao, China). LB broth, LB agar, D-glucose, ampicillin sodium, kanamycin, and non-fat powdered milk were purchased from Solaibao Technology Co., Ltd. (Beijing, China). The 0.22 μm PES needle filter was sourced from Millipore (Burlington, MA, USA).

The blocking solution (3% PBSTM) was fabricated by precisely weighing 0.6 g of skimmed milk powder and dissolving it in 20 mL of PBST solution. The lysis buffer (NPI-10) was composed of 50 millimolar NaH_2_PO_4_, 300 millimolar NaCl, and 10 millimolar imidazole, with a pH value of 7.4. PEG/NaCl: 200 g of PEG6000 and 146.1 g of NaCl were weighed out and dissolved in 1 L of ddH_2_O.

### 2.2. Alpaca Immunization and Serum Titer Test

An adult male alpaca was sourced from Guangzhou Rongchuan Zoo for the study. The immunogen, ZEN-BSA (200 μg), was mixed and emulsified with an equal volume of Freund’s complete adjuvant. The emulsion was administered subcutaneously near the neck lymph nodes. Booster immunizations, consisting of 100 μg ZEN-BSA emulsified in Freund’s incomplete adjuvant, were administered biweekly [28]. Post-immunization, the alpaca was monitored for 30 min to ensure physiological stability. Blood samples (5 mL) were collected pre-immunization and on the seventh day following each immunization. Alpaca immunization and blood collection were conducted at Guangzhou Rongchuan Zoo. Samples were coagulated at room temperature for 2 h and centrifuged at 3000× *g* for 5 min, and the resulting serum was stored at −20 °C. Antibody titers were evaluated via indirect ELISA [33]. Briefly, 96-well plates were coated with 2 μg/mL ZEN-OVA. Serially diluted alpaca serum served as the primary antibody, with pre-immune serum and 1× PBS buffer as negative and blank controls. Goat anti-alpaca IgG-HRP conjugate was used as the secondary antibody. After washing with PBST five times, TMB substrate was added and incubated at 37 °C in the dark for 10 min. The reaction was terminated with a TMB stop solution, and optical density (OD450 nm) was measured. Serum titer was defined as the maximal dilution yielding detectable antigen–antibody reactivity.

### 2.3. Construction of the Phage Display Nanobody Library

Upon reaching peak antibody titers, immunization was discontinued. Venous blood (30 mL) was collected from the alpaca using EDTA-anticoagulated vacuum tubes. Peripheral blood lymphocytes (PBLs) were isolated by density gradient centrifugation. Total RNA was extracted from PBLs using Trizol reagent, and complementary DNA (cDNA) was synthesized via reverse transcription with random hexamer primers. A two-step nested PCR strategy was employed to amplify nanobody gene fragments [34]. The primary PCR utilized primers CALL001 (GTC CTG GCT GCT CTT CTA CAA GG) and CALL002 (GGT ACG TGC TGT TGA ACT GTT CC), which targeted the leader and constant region, were used under the following conditions: 95 °C for 3 min (initial denaturation); 30 cycles of 94 °C for 1 min (denaturation), 57 °C for 1 min (annealing), and 72 °C for 45 s (extension); followed by a final elongation at 72 °C for 7 min. The resulting 700 bp products were gel-purified and served as templates for the secondary PCR. The secondary PCR used primers VHH-F (CAT GCC ATG ACT GTG GCC CAG GCG GCC CAG KTG CAG CTC GTG GAG TC) and VHH-R1 (CAT GCC ATG ACT CGC GGC CGG CCT GGC CAT GGG GGT CTT CGC TGT GGT GCG)/VHH-R2 (CAT GCC ATG ACT CGC GGC CGG CCT GGC CGT CTT GTG GTT TTG GTG TCT TGG G), designed to anneal to framework region 1 (FR1) and the hinge region, and introduced *Sfi*I restriction sites. Cycling conditions included: 94 °C for 3 min; 24 cycles of 98 °C for 10 s, 55 °C for 15 s, and 72 °C for 30 s; with a final extension at 72 °C for 10 min. The phagemid vector pComb3XSS and PCR products were digested with *Sfi*I at 50 °C. To minimize self-ligation, the vector was dephosphorylated. The digested products were ligated at a 1:3 molar ratio and incubated overnight at 16 °C. The recombinant phagemids were transformed into chemically competent *E. coli* TG1 cells and cultured overnight, yielding the primary nanobody library [35]. Twenty clones were randomly selected and screened by colony PCR to assess the insertion rate. Clones containing appropriately sized inserts were cultured in 2 mL of LB-Amp medium and incubated for 12–16 h at 37 °C and 200 rpm. Bacterial cultures were submitted for Sanger sequencing [36] to evaluate the library’s diversity. Cells from 1 mL of LB culture were plated on LB/Amp-Glu agar. Clones were harvested with a sterile cell scraper, resuspended in 20% (*v*/*v*) sterile glycerol, snap-frozen in liquid nitrogen, and stored at −80 °C.

The primary nanobody library was inoculated into 100 mL of 2× YT-Amp-Glu medium and incubated at 37 °C with shaking at 200 rpm for 2 h until the OD600 reached approximately 0.5. M13KO7 helper phage was added at a multiplicity of infection (MOI) of 10:1, followed by gentle agitation and static incubation at 37 °C for 30 min to facilitate phage adsorption. The culture was then shaken (200 rpm) for an additional 60 min at 37 °C to promote bacterial recovery. The culture was centrifuged at 22 °C and 2800× *g* for 10 min. The pellet was resuspended in 80 mL of 2× YT-Amp-Kana medium and incubated overnight at 37 °C with shaking at 200 rpm to amplify phage particles. The suspension was centrifuged again for 15 min at 4 °C and 3200× *g*, and the supernatant was collected. Phage particles were precipitated by adding a 1/4 volume of PEG/NaCl solution, then resuspended in PBS and filtered through a 0.22 µm filter to produce the phage display nanobody library. The library was supplemented with 20% (*v*/*v*) glycerol, snap-frozen in liquid nitrogen, and stored at −80 °C. To determine library titer, ten-fold serial dilutions of 1 μL of the library were prepared, and phage titers were quantified to assess library diversity.

### 2.4. Screening and Identification of Anti-ZEN Phage Display Nanobodies

An affinity-based biopanning method was employed to enrich phage clones with high affinity for ZEN from the phage display nanobody library [37]. The protocol comprised four rounds of panning, utilizing ZEN-OVA as the antigen, with sequential reductions in coating concentrations (10, 5, 2.5, and 1 µg/mL). Desorption wells were alternately coated with 2% nonfat powdered milk or 2% bovine serum albumin (BSA) to minimize nonspecific binding. The initial two rounds used 0.1 M glycine-HCl for eluting bound phages, while the latter two rounds employed competitive elution with ZEN liquid standard at concentrations of 100 and 50 ng/mL, respectively. Amplification of eluted phages preceded each subsequent round. Phage enrichment was quantified by comparing input and recovered titers, with the recovery rate determined by dividing the recovered phage titer by the input phage titer. Enrichment was ascertained by comparing the recovery rates between consecutive rounds. This systematic biopanning successfully led to the enrichment of phage clones with high ZEN-binding affinity.

To ascertain the binding activity of the clones against zearalenone (ZEN), a selection of 72 individual phage clones was randomly made from the elution plates following the second, third, and fourth rounds of panning [38]. These clones were infected with the helper phage M13KO7, and their binding affinity for ZEN was assessed using an indirect competition phage enzyme-linked immunosorbent assay (ELISA) [33]. The assay protocol commenced with coating a 96-well microplate with the antigen ZEN-OVA at a concentration of 1 µg/mL and incubating it overnight at 4 °C. Each phage clone was tested in duplicate wells to ensure the reproducibility of the results and to prevent cross-contamination. Following washing, 300 µL of 3% PBSTM blocking solution was added, and the plate was incubated for 1 h at 37 °C. After removing the blocking solution and washing three times with 300 µL of PBST, one well received a mixture containing 50 µL of a 10-fold diluted phage supernatant and 50 µL of a 500 ng/mL ZEN solution, while the other well received 50 µL of a 10-fold diluted phage supernatant and 50 µL of PBS buffer. A 100 µL aliquot of PBS served as a blank control, and the plate was incubated for 1 h at 37 °C. The plate was washed, and bound phages were detected using an anti-M13 HRP-conjugated antibody (1:5000 dilution, 1-h incubation). Thereafter, 100 μL of TMB chromogenic substrate solution was added to each well, and the plate was incubated at 37 °C for 10 min in the dark. TMB substrate was halted with stop solution, and absorbance at 450 nm was measured. Phage clones that demonstrated a robust competitive blocking effect and high-affinity binding to ZEN were selected for Sanger sequencing to confirm nanobody gene sequences.

### 2.5. Molecular Recognition Mechanism of Nanobody and Zearalenone

The nucleotide sequence of the nanobody was translated into its corresponding amino acid sequence using the Expasy-Translate tool [39]. Subsequent homology modeling was performed via the Swiss model, where sequence alignment against the Protein Data Bank (PDB) was conducted using BLAST to identify templates with maximal homology. A three-dimensional structural model was generated using the highest-homology template. Model validation included stereochemical quality assessment via a Ramachandran plot (generated with PDBsum [40]), which evaluates the phi (φ) and psi (ψ) dihedral angles of residues to ensure their placement within energetically favorable regions. The ZEN (CID: 5281576) molecular structure was retrieved from the PubChem database [41]. Molecule docking simulations were performed using Autodock Tools [42]. Preparatory steps included the ablation of water molecules from the nanobody structure, addition of hydrogen atoms to the nanobody and setting it as a macromolecule, addition of hydrogen atoms to ZEN and setting it as a ligand with auto-distributed charge, detection, and selection of torsion bonds in ZEN. A docking box was established to encompass the protein, with ZEN positioned exteriorly. AutoGrid4 calculated atomic affinity potentials, electrostatic interactions, and a desolvation energy map. AutoDock4 executed 50 independent docking runs using the Lamarckian genetic algorithms. The conformation with the lowest binding free energy was selected as the optimal pose. Final visualizations and interaction analyses (hydrogen bonds, hydrophobic contacts) were performed in the Pymol [43], identifying critical nanobody residues involved in ZEN binding and elucidating atomic-level interaction mechanisms.

### 2.6. Expression and Purification of Anti-ZEN Nanobody

The prokaryotic expression system of *Escherichia coli* was employed for the expression and preparation of the nanobody. Since the nanobody was displayed on the phage surface, the plasmid from the positive phage clone was extracted and transformed into the non-inhibitory *E. coli* TOP 10F’ competent cells for independent expression. The mixture was gently mixed by pipetting and incubated on ice for 25 min to facilitate DNA uptake. To enhance transformation efficiency, a 45 s heat shock at 42 °C was applied, followed by immediate cooling on ice for 2 min. Subsequently, 700 µL of LB liquid medium was added to the 1.5 mL microcentrifuge tube and mixed gently. The mixture was then revived for 1 h at 37 °C and 250 rpm. Afterward, the mixture was centrifuged at room temperature at 2300× *g* for 1 min. Approximately 100 µL of the supernatant was retained, appropriately diluted, and plated onto LB-Amp agar plates. The plates were incubated overnight at 37 °C.

Individual monoclonal colonies from the transformation plates were inoculated into 3 mL of LB-Amp liquid medium and cultured for 12–16 h at 37 °C with shaking at 250 rpm. The culture was then transferred to 100 mL of LB-Amp medium at a 1:100 dilution and incubated at 37 °C with shaking at 150 rpm for 5 h until the OD600 reached approximately 0.6. Protein expression was induced with 1 mM IPTG, followed by incubation for 8 h at 30 °C and 150 rpm. The bacterial cells were harvested by centrifugation at 6000× *g* and 4 °C for 5 min. The pellet was resuspended in 630 µL of lysis buffer NPI-10, incubated on ice for 30 min [35], and vortexed briefly every 10 min. Cell disruption was achieved via ice bath sonication (10 min total, 2 s pulse with 2 s intervals). The lysate was centrifuged at 4 °C and 5000× *g* for 10 min, and the supernatant was retained after filtration through a 0.22 µm filter.

The nanobody was purified using nickel-nitrilotriacetic acid (Ni-NTA) affinity chromatography. The clarified lysate was loaded onto the column at a flow rate of 1 mL/min, and the eluate was collected. A gradient of imidazole concentrations from 10 to 200 mM was applied in 1 mL fractions to elute the bound protein. Eluted fractions were analyzed by SDS-PAGE, and those containing the target protein were identified by Coomassie brilliant blue staining and gel imaging. The purified nanobody was collected, transferred to a dialysis bag with a 3500 Da molecular weight cutoff, and dialyzed against PBS buffer. The nanobody was then concentrated using a 3000 Da ultrafiltration centrifuge tube [44]. SDS-PAGE and Western blot analysis confirmed the presence of the nanobody. For Western blotting, after electrophoresis and membrane transfer, the membrane was blocked with 5% skim milk powder for 2 h. The membrane was then incubated overnight with a 1:2000 dilution of Anti-6× His tag antibody. Following three washes with PBST, the membrane was incubated for 1 h with a 1:2000 dilution of HRP-conjugated goat anti-rabbit IgG (H + L) secondary antibody. The membrane was finally washed, developed with the appropriate substrate, and imaged to detect the nanobody band.

### 2.7. Determining the Specificity and Binding Affinity of Anti-ZEN Nanobody V44

To assess the specificity of the nanobody, an indirect ELISA was conducted. This method was chosen for its capacity to measure selective binding while concurrently detecting potential cross-reactivity with carrier proteins. The experimental procedure involved coating a 96-well microplate with ZEN-OVA, ZEN-BSA, OVA, and BSA proteins at 2 μg/mL, using PBS as a negative control [37]. Following overnight incubation at 4 °C, the wells were blocked with a blocking solution at 37 °C for 2 h. Subsequently, the nanobody V44 with a dilution of 1:2000, along with an anti-His-HRP enzyme-labeled secondary antibody, was added. After washing, a TMB chromogenic substrate solution was added, and the plate was incubated for 15 min at 37 °C in darkness. The reaction was terminated, and optical density measurements were taken to determine antigen specificity.

For binding affinity analysis, an indirect competition ELISA was conducted [45]. The microplate was coated with the target antigen, and the optimal concentrations of both the coated antigen and the nanobody were determined by checkerboard titration. Serial dilutions of the coating antigen (500-, 1000-, 2000-, 4000-, and 8000-fold) and the nanobody (200-, 400-, 800-, 1000-, and 2000-fold) were tested in parallel using blank and inhibitory wells. Optimal parameters were defined based on achieving blank well absorbance within the target range (0.8–1.0) and maximal inhibition rates. A standard curve was created using the selected concentrations, and the IC_50_ value was calculated, with lower values indicating higher detection sensitivity. The practical detection range between IC_20_ and IC_80_ inhibitory concentrations was established to ensure reliable measurements.

### 2.8. Statistical Analysis

Statistical analysis and graph generation were performed using OriginPro 2024 (OriginLab, Northampton, MA, USA) and GraphPad Prism software (version 8.0). Data from phage ELISA experiments, the specificity and binding affinity assessments of the anti-ZEN nanobody, are presented as mean ± standard deviation (S.D.) and represent three independent experimental replicates. The specificity of the anti-ZEN nanobody was assessed through a one-way analysis of variance (ANOVA), with statistical significance defined as *p* < 0.05. For icELISA, standardized inhibition curves were generated using nonlinear regression analysis (curve fitting), using a four-parameter logistic dose-response-inhibition model.

## 3. Results

### 3.1. Serum Antibody Titer Test

As depicted in Figure S1, the serum titer reached 32,000 after the fourth immunization, satisfying the minimum titer level necessary for the construction of the library. Consequently, 30 mL of venous blood was collected from the alpaca after the fourth immunization for subsequent experimentation.

### 3.2. VHH Gene Amplification

**Figure 1:**
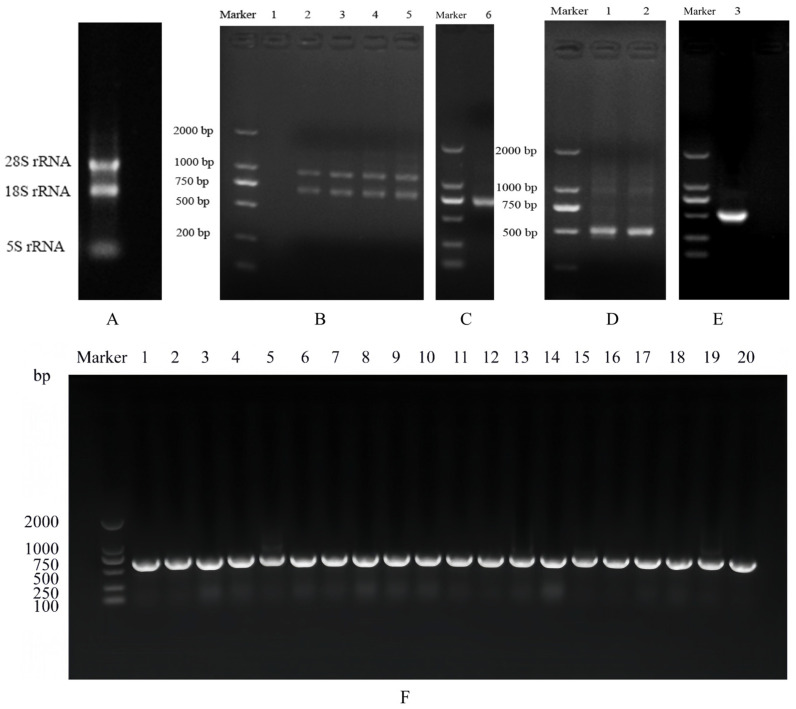
Library construction. (**A**) Total RNA extracted; (**B**) lanes 1 to 5: 0, 0.5, 1, 2, and 4 µL cDNA templates; (**C**) lane 6: gel recovery of PCR-1 products; (**D**) lane 1: VHH-F/VHH-R1 amplification product, and lane 2: VHH-F/VHH-R2 amplification product; (**E**) lane 3: gel recovery of PCR-2 products; and (**F**) colony PCR identification of the primary nanobody library.

Approximately 1.17 × 10^7^ peripheral blood lymphocytes were isolated and subjected to total RNA extraction. The extracted RNA demonstrated high purity, with a concentration of 336.484 µg/mL and an OD260/OD280 ratio of 2.021, confirming minimal DNA or protein contamination. The integrity of the RNA was further validated via 1% agarose gel electrophoresis, as presented in Figure 1A, which revealed sharp, distinct bands corresponding to 28S, 18S, and 5S ribosomal RNA subunits. The VHH gene was amplified via nested PCR using cDNA synthesized from the extracted RNA. Initial PCR optimization tested varying cDNA template volumes (0, 0.5, 1, 2, and 4 μL), culminating in the amplification of two specific bands: a 1000 bp band corresponding to the conventional antibody product and a 700 bp band corresponding to the heavy chain antibody product. Optimal amplification was attained with 2 μL of cDNA, which was consequently selected for further PCR amplification. The 700 bp fragment was gel-purified and utilized as the template for the second round of nested PCR. Two pairs of primers, VHH-F1/VHH-R1 and VHH-F2/VHH-R2, were employed to amplify a 500 bp nanobody gene fragment. The final PCR product was gel-purified.

### 3.3. Identification of Primary Nanobody Library

**Figure 2:**
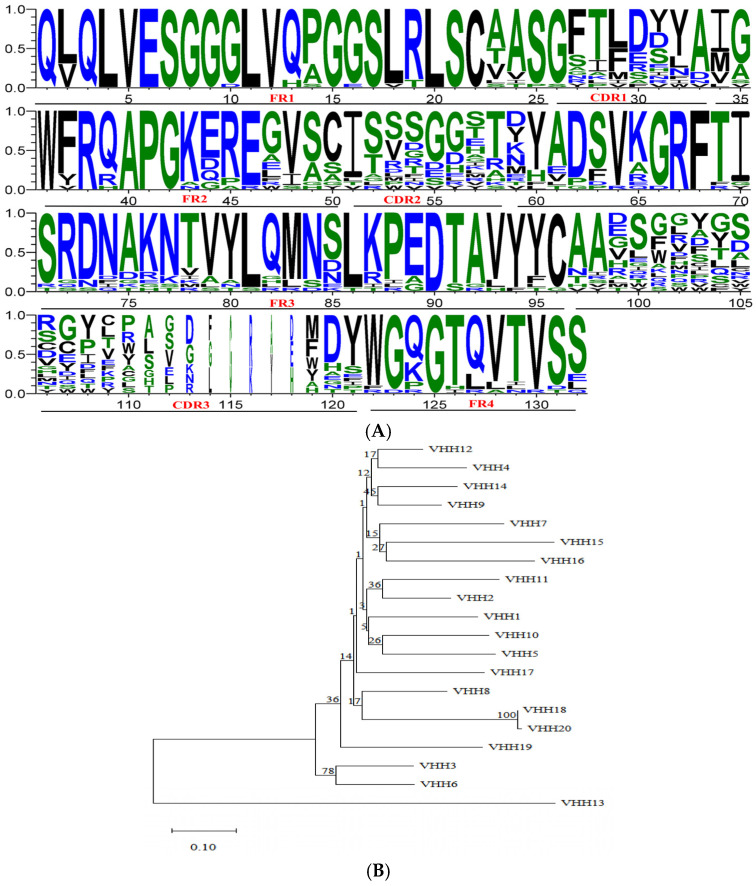
Identification library. (**A**) Amino acid sequence alignment analysis of the primary nanobody library. DNA sequences were translated into amino acid sequences using ExPASy, and sequence conservation and variability were analyzed using WebLogo to generate frequency distribution profiles. (**B**) Phylogenetic tree of 20 nanobody sequences. A neighbor-joining phylogenetic tree was constructed using MEGA 11, with branch support assessed by the bootstrap method (1000 replications).

Twenty clones were randomly selected and analyzed by PCR to confirm nanobody gene insertion. As illustrated in Figure 1F, a distinct band ranging from 500 to 750 bp was observed in all clones, indicating successful gene incorporation with a 100% positive rate. To evaluate sequence diversity, Sanger sequencing was performed on these clones. Figure 2A,B depict the sequence features of the framework regions (FR1, FR2, FR3, FR4) and the complementarity determining regions (CDR1, CDR2, CDR3). Among the twenty clones, eighteen exhibited unique DNA sequences, while two clones shared identical sequences. Notably, the CDR3 region demonstrated 95% amino acid sequence diversity, demonstrating a high level of sequence diversity within the immunized nanobody library. The estimated capacity of the primary nanobody library was 1.11 × 10^7^ cfu.

### 3.4. Library Biopanning

The primary nanobody library was amplified utilizing M13KO7 helper phage and subsequently purified with PEG-NaCl, yielding a phage display nanobody library with a titer of 1.0 × 10^11^ pfu/mL. Four rounds of panning were performed to identify phage clones exhibiting high-affinity, selective binding to ZEN. Table 1 summarizes the experimental parameters and outcomes for each panning round. By the fourth panning round, the phage recovery rate increased significantly, achieving a 479-fold enhancement, which signified the effective enrichment of ZEN-specific binding phages.

### 3.5. Identification of Positive Phage Clones

**Figure 3:**
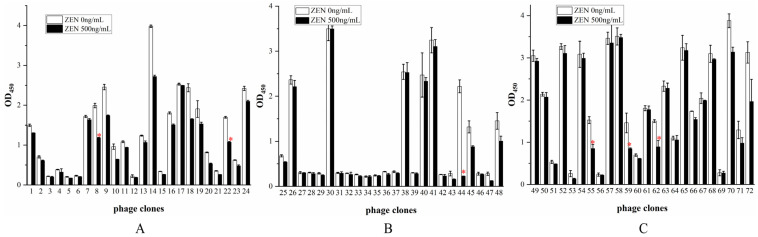
Indirect competition phage ELISA screening of phage clones: the (**A**) second round of panning; (**B**) third round of panning; and (**C**) fourth round of panning. Data are presented as means ± S.D. (*n* = 3). Clones (V8, V22, V44, V55, V59, and V62) with ELISA signal greater than 1.0 in the absence of ZEN and with >40% signal inhibition in the presence of ZEN were selected for further analysis and marked with “*” in the image.

Seventy-two individual phage clones were randomly selected from the eluates of the second, third, and fourth rounds of panning for further analysis. Following culture expansion, clones were evaluated using an indirect competition phage ELISA. As illustrated in Figure 3, the absorbance values of the standard wells of 40 phage clones at OD450 nm were greater than 1, accounting for 55.56%; the majority of the clones demonstrated binding to the ZEN-OVA antigen. A subset of clones showed inhibited binding upon the addition of free ZEN standard solution. The six phage clones that displayed the higher inhibition rates were identified as V8, V62, V59, V22, V55, and V44, with respective inhibition rates of 41.57%, 41.79%, 43.17%, 44.33%, 45.47%, and 91.56%.

**Figure 4:**
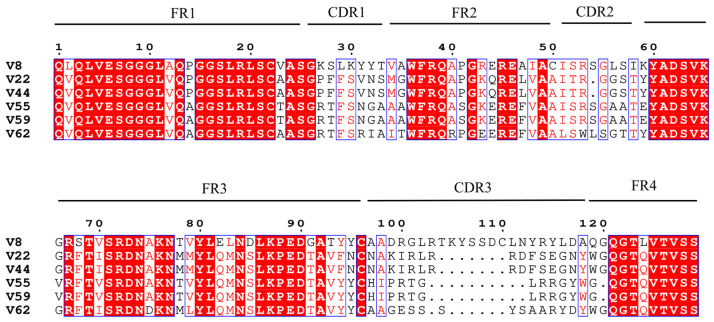
Amino acid sequence alignment analysis of anti-ZEN VHH. Significant variability was observed in the CDR3 region, which is a critical determinant of epitope recognition and functional activity within nanobodies. Specifically, the CDR3 region length differed across variants: V8 exhibited 22 amino acids, while V44, V55, and V62 displayed shorter sequences of 15, 12, and 15 amino acids, respectively.

Sanger sequencing was employed to determine the DNA sequences of these clones, which were subsequently translated into amino acid sequences for alignment, as depicted in Figure 4. Six clones corresponded to four unique amino acid sequences: V8, V44 (including V22), V55 (including V59), and V62. Notwithstanding that phage clones V44 and V22 possess the same sequence, the phage ELISA outcomes evinced discrepant activities vis-à-vis ZEN. This discrepancy may reflect variations in the quantum of phages, which influences nanobodies’ display on the phage surface. Higher phage numbers increase nanobody presentation and enhance binding activity. All sequences possessed a complete nanobody structural framework with high regional homology. In the FR2 region, three hydrophobic residues (V, G, and L) were mutated to three hydrophilic residues (F37, E44, and R45), enhancing the water solubility of nanobody V8, V44, V55, and V62 [46]. Additionally, the presence of charged amino acid residues (D62, K65, R67, R72, K76, and E89) in the FR3 region helps to reduce aggregation and promote the reversible folding, thermal stability, and solubility of Nbs, making them superior to traditional antibodies [47]. Structural stability was reinforced by conserved disulfide bonds: V44, V55, and V62 each harbored two cysteines forming a single FR1-FR3 band, whereas V8 contained four cysteines, forming two internal disulfide bonds located in the FR1-FR3 and FR2-CDR3 regions, respectively [46]. To assess ZEN-binding affinity, the nanobodies’ 3D structures were computationally modeled and docked with ZEN, enabling the analysis of the molecular recognition mechanism and identification of critical binding residues.

### 3.6. Homology Modeling and Molecular Docking

**Figure 5:**
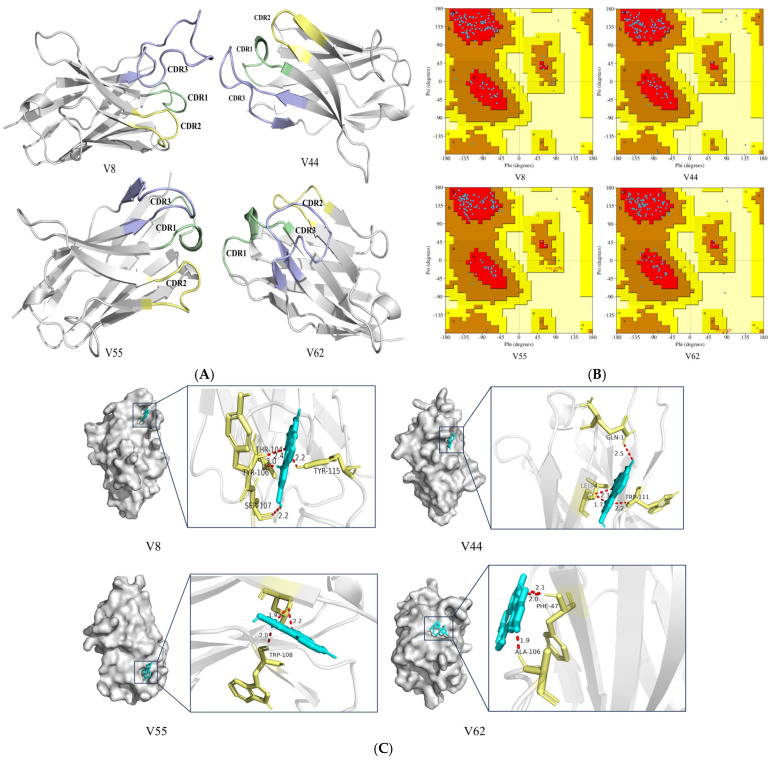
Three-dimensional structure simulation and docking analysis of VHH. (**A**) Homology-modeled 3D structure of V8, V44, V55, and V62. FRs are shown as anti-parallel β-sheets (gray), with CDRs colored as follows: CDR1 (pale green), CDR2 (pale yellow), and CDR3 (light blue). (**B**) Ramachandran plot validation of model quality, with glycine and proline residues excluded. Regions are defined as the most favored [A, B, L], additional allowed [a, b, l, p], generously allowed [~a, ~b, ~l, ~p], and disallowed. All models met high-quality criteria, with 90% or more of the residues in the most favored regions. (**C**) ZEN binding mode predicted by semi-flexible docking (Autodock, Lamarckian genetic algorithm/LGA). Optimal conformations (50 cycles) revealed stable ligand–receptor complexes with binding energies that were all below 5 kJ/mol. The green part represents ZEN and the yellow part represents the amino acid residues of the nanobody.

The V8, V44, V55, and V62 gene sequences were translated into 129, 121, 118, and 122 amino acid sequences (excluding 6× His tags and HA tags), respectively [39]. Structural modeling utilized the following SMTL templates: 5hdo.4 (V8), 7o06.1 (V44), 4tvs.1 (V55), and 5iml.1 (V62). As depicted in Figure 5A, all models possess a cylindrical configuration, with the FR characterized by reverse-parallel β-folding and the CDR dominated by α-helices and irregular coiling. Notably, the CDR forms elevated structures on the surface, potentially being associated with the single-domain structure of nanobodies. Model reliability was validated via Ramachandran plot analysis [40]. The Ramachandran plot (Figure 5B) shows that 91% of the amino acid dihedral angle coordinate points of V8 are in the most favored regions, with an additional 9% falling within the allowed regions. Similarly, 98% of the amino acid dihedral angle coordinate points of V44 are in the most favored regions, with 2% in the allowed regions. For V55, 95% of the amino acid dihedral angle coordinate points are in the most favored regions, 4% in the allowed regions, and 1% in the generously allowed regions. Finally, for V62, 93.4% of the amino acid dihedral angle coordinate points are in the most favored regions, 5.7% in the allowed regions, and 0.9% in the generously allowed regions. These distributions indicate that these 3D models are of high quality and are structurally plausible.

As shown in Figure 5C, molecular docking analysis revealed that ZEN binds to nanobodies V8, V44, V55, and V62 in a characteristic “side-on” binding mode, aligning with previous studies of antigen insertion into lateral/apical pockets of antibody structures [48]. The interaction was chiefly mediated by hydrogen bonding and hydrophobic interactions [43]. In V8, hydrogen atoms on ZEN form bonds with the oxygen atoms of THR104 and SER107, while its oxygen atoms engage TYR115 via a double hydrogen bond. Three amino acid residues (GLN1, LEU4, and TRP111) in V44 are crucial for binding ZEN. V55 relied on TRP108 for hydrogen bonding, whereas V62 utilized PHE47 and ALA106. Critically, all hydrogen bonding occurred within the CDR3 region, identifying it as the central ZEN recognition domain. These structural insights, combined with molecular dynamics simulation, elucidate nanobody-ZEN binding mechanisms and provide a roadmap for targeted mutagenesis of binding residues.

### 3.7. Purification and Identification of Anti-ZEN VHH

To enable independent expression, the plasmid was transformed into the non-inhibitory *E. coli* TOP 10F’ host strain. Phage-V44, with the highest inhibition rate, was chosen for subsequent expression and purification.

**Figure 6:**
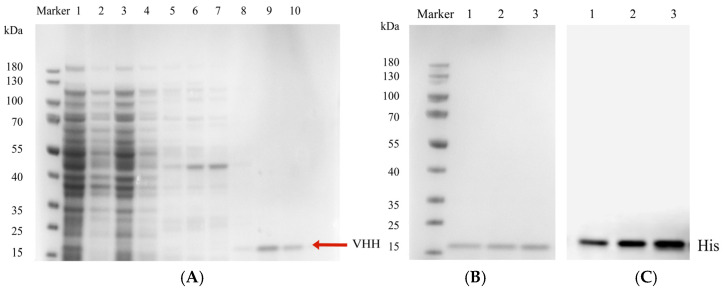
Expression and purification results of nanobody V44. (**A**) For extraction and purification of the nanobody, lane 1 represents the total protein in the original sample, while lane 2 shows the flow-through eluate. Lanes 3 to 10 manifest the eluate from 10 mM, 20 mM, 30 mM, 40 mM, 50 mM, 100 mM, 100 mM, and 200 mM imidazole solutions, respectively. The flow-through eluate was ascertained to be predominantly devoid of target proteins, signifying that the preponderance of target proteins were successfully bound to the Ni column. This also intimates that high concentrations of imidazole (100 mM and 200 mM) were requisite to competitively elute the nanobody. (**B**) SDS-PAGE images of a nanobody; lanes 1–3 represent different loading amounts of nanobody V44. (**C**) Western blot images of a nanobody; lanes 1–3 represent different loading amounts of nanobody V44.

The bacterial culture was induced with 1 mM IPTG and incubated at 30 °C with shaking at 200 rpm for 8 h to express the nanobody. Following ultrasonic lysis in an ice bath, the lysate was purified using nickel affinity chromatography. Eluates from the imidazole gradient were analyzed via SDS-PAGE, as presented in Figure 6A. The target protein was successfully eluted at the second 100 mM and the 200 mM imidazole concentrations, demonstrating high purity with minimal contaminants. The eluates were collected under two distinct conditions, then transferred into 3500 Da MWCO dialysis membranes for dialysis against PBS buffer and concentrated with a 3000 Da ultrafiltration device. The purity and identity of the nanobody were confirmed through SDS-PAGE and Western blot analyses. As depicted in Figure 6B,C, the purified nanobody exhibited a molecular weight of approximately 17 kDa, consistent with its expected molecular weight. A distinct band on the Western blot indicated specific binding to the anti-6× His rabbit monoclonal antibody, confirming the successful isolation and preparation of the nanobody.

### 3.8. Validation of Specific Anti-ZEN VHH

**Figure 7:**
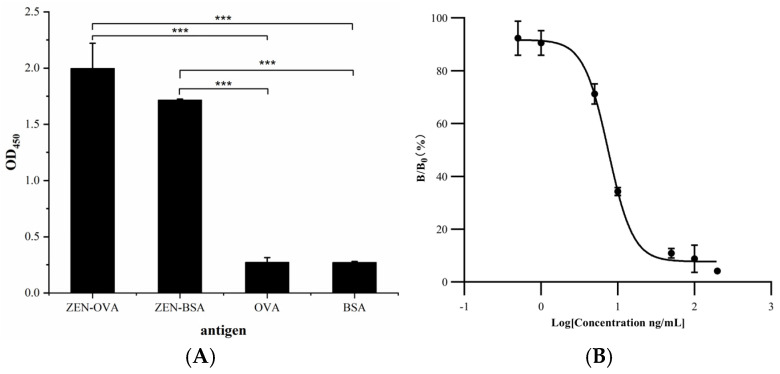
Specificity and Affinity of anti-ZEN VHH. (**A**) The binding specificity of purified nanobodies to ZEN was detected by indirect ELISA. The results are presented as mean absorbance values at OD450 nm ± SD (*n* = 3). Statistical analysis was performed with one-way ANOVA (*** *p*< 0.001). (**B**) The standard inhibition curve of the icELISA for zearalenone, an IC_50_ value of 7.55 ng/mL, a detection range of 4.52–12.62 ng/mL (IC_20_–IC_80_ inhibition concentration), and a correlation coefficient of R^2^ = 0.99. The analysis of the absorbance of blank wells and the inhibition rates of corresponding inhibitory wells helped determine the optimal conditions for the working curve development.

The specificity of nanobody V44 for ZEN was assessed via indirect ELISA. ZEN-BSA and ZEN-OVA were chosen as target antigens owing to their molecular structure similarity to the target compound. Meanwhile, BSA and OVA were used as negative control antigens, with PBS as a blank control. As shown in Figure 7A, the results revealed that the nanobody V44 exhibited a high degree of specific binding with the ZEN-BSA and ZEN-OVA antigens, while demonstrating minimal binding to the control antigens BSA and OVA. These comparative data established the high specificity of the anti-ZEN VHH.

As presented in Table 2, both the nanobody and the coated antigen were diluted 2000-fold using ZEN-OVA, as this dilution ratio provided the maximal assay sensitivity during preliminary optimization experiments. The resulting working curve, illustrated in Figure 7B, exhibited an IC_50_ value of 7.55 ng/mL, which is comparable to or superior to previously reported antibody-based detection methods for similar compounds [22]. A linear detection range of 4.52–12.62 ng/mL (IC_20_–IC_80_ inhibition concentration) and a correlation coefficient of R^2^ = 0.99 indicated excellent assay performance. These findings confirm V44’s high specificity and affinity for ZEN, supporting its utility in immunoassay development for mycotoxin detection.

## 4. Discussion

Our study utilizes an immune phage display library generated from an alpaca immunized with the ZEN-BSA antigen, allowing for the direct selection of high-affinity nanobodies against the target antigen, ZEN. The serum antibody titer of the alpaca plateaued at 32,000 following the fourth immunization, with no subsequent increase. Although this immunization protocol elicited an effective immune response, the peak antibody titer, as reported in a previous study [28], was only achieved after six immunizations. This discrepancy may stem from individual variability in alpaca health status or immune responses. Therefore, it is suggested that multiple alpacas be immunized, and peripheral blood collected for further experimental procedures to account for intersubject differences. Following the third immunization, localized inflammation occurred at the injection site, likely a result of Freund’s adjuvant. Although Freund’s adjuvant is widely used to prolong antigen release, amplify adaptive immunity, and induce the production of IgG antibodies [49], it is also associated with an influx of white blood cells and subsequent inflammation at the injection site. Despite its adverse effects, no suitable alternatives are available. To reduce adverse reactions, dispersing the injection site during subsequent immunizations is recommended.

The phagemid vector pComb3XSS encompasses only a subset of the phage’s genetic material and cannot independently complete the assembly of phage particles. To enable replication and packaging, the helper phage M13KO7 must be introduced, as it provides essential protease and coat proteins [50]. Utilizing the helper phage M13KO7, the primary nanobody library was rescued and amplified, followed by purification with PEG-NaCl, yielding a phage display nanobody library with a titer of 1.0 × 10^11^ pfu/mL. High-affinity phage clones were isolated through a process of acid elution and competitive elution. Progressive increases in stringency during acid elution preferentially enriched high-affinity antibodies, while competitive elution further enhanced specificity by displacing weakly bound phages [51]. Given that the antigen in use was ZEN-BSA, the library inherently contained both anti-ZEN and anti-BSA nanobodies. To mitigate nonspecific binding, the library was alternately incubated with 2% BSA and 2% nonfat powdered milk during screening, thereby suppressing BSA-targeting clones and enriching ZEN-specific nanobodies. Indirect competitive phage ELISA was employed to confirm positive phage clones and optimize the concentrations of the competitive standards. In the fourth (and most stringent) panning round, a greater number of clones from the elution plate was selected for analysis, thereby increasing the probability of isolating highly specific phage display nanobodies.

Wang et al. [52] conducted a study to identify the optimal antibody/antigen combination for a heterologous icELISA. The immunogen ZEN-BSA was synthesized using two methods: amino glutaraldehyde (AGA) and amino diazotization (AD). In contrast, the coating antigens ZEN-OVA were synthesized via five distinct methods: oxime active ester (OAE), formaldehyde (FA), 1,4-butanediol diglycidyl ether (BDE), AGA, and AD. Utilizing the mAb 2B6, the researchers developed an icELISA that demonstrated an IC_50_ of 8.69 μg/L. A standard inhibition curve for zearalenone established in this study had an IC_50_ of 7.55 ng/mL and a detection range of 4.52–12.62 ng/mL (IC_20_–IC_80_ inhibitory concentrations), with a correlation coefficient of R^2^ = 0.99. These results are comparable to those of previously reported antibody-based assays for similar compounds. Further optimization of experimental parameters, such as blocking agents, pH, and ionic strength, could further enhance the assay’s sensitivity.

Current research efforts are concentrated on the development and characterization of anti-ZEN nanobodies, which show significant potential for facilitating the rapid detection of ZEN in food and feed products. However, further validation in real samples, such as animal feed and human food products, is a necessity. In order to achieve this objective, future studies should encompass a comprehensive approach, including real sample testing, spiking experiments, comparative analyses with established methods, and extensive field testing. Depending on the availability of resources and samples, explore the feasibility of conducting field tests in collaboration with local agriculture and food safety authorities to assess the practical applicability of the nanobodies in real-world settings. This advancement would provide a scientific foundation for assessing ZEN contamination levels, performing risk assessments, and identifying and removing contaminated products before they reach consumers, thereby reducing potential ZEN exposure risks. The implications of this work are far-reaching and multifaceted, encompassing the prevention of ZEN-associated risks, the enhancement of food safety measures, and the comprehensive safeguarding of both animal and human health.

## 5. Conclusions

In this study, we used ZEN-BSA to immunize alpacas with a titer of 1:32,000. We successfully constructed a primary nanobody library with a capacity of 1.11 × 10^7^ cfu and obtained an anti-ZEN phage-displayed nanobody library with a capacity of 1.0 × 10^11^ pfu/mL through rescue and amplification. After four rounds of biopanning, we obtained four specific ZEN-binding phage display nanobodies. The anti-ZEN nanobody (V44) was expressed and purified considering inhibition rate values. The specificity and affinity of the nanobody were also verified. We established a working curve for ic-ELISA for ZEN, which showed an IC_50_ value of 7.55 ng/mL and a correlation coefficient of R^2^ = 0.99.

## Figures and Tables

**Table 1:** Phage enrichment during four rounds of biopanning.

Round	ZEN-OVA (μg/mL)	Input (pfu/mL)	Output (pfu/mL)	Recovery Rate	Enrichment
1	10.00	1.00 × 10^11^	5.55 × 10^4^	5.55 × 10^−7^	/
2	5.00	8.70 × 10^11^	1.04 × 10^8^	1.20 × 10^−4^	216.22
3	2.50	1.21 × 10^11^	1.24 × 10^7^	1.02 × 10^−4^	0.85
4	1.25	7.00 × 10^11^	1.86 × 10^8^	2.66 × 10^−4^	2.66

**Table 2:** Optimal working concentration of anti-ZEN nanobody V44 and coated antigen.

ZEN-OVA	Anti-ZEN VHH									
	200		400		800		1000		2000	
	0	1	0	1	0	1	0	1	0	1
500	1.65	1.37	1.23	0.94	0.94	0.50	0.88	0.50	0.72	0.36
1000	1.82	1.54	1.48	1.13	1.33	0.89	1.31	0.84	1.04	0.61
2000	1.70	1.44	1.43	1.06	1.22	0.80	1.22	0.90	1.03	0.49
4000	1.37	1.24	1.11	0.73	0.83	0.47	0.77	0.44	0.64	0.26
8000	1.24	1.08	1.10	0.80	1.01	0.56	0.50	0.32	0.42	0.27

Note: 0 indicates control wells in the microplate; 1 indicates inhibition wells in the microplate (concentration of ZEN 100 ng/mL).

## Data Availability

The datasets used or analyzed during the current study are available from the corresponding author on reasonable request.

## Supplementary Materials

The following supporting information can be downloaded at https://www.mdpi.com/article/10.3390/cimb47030157/s1.
