# Supplementary material for: Development of an Anti-Zearalenone Nanobody Phage Display Library and Preparation of Specific Nanobodies
Source: Curr Issues Mol Biol. 2025 Feb 27;47(3):157. doi: 10.3390/cimb47030157 (PMC11940844; doi:10.3390/cimb47030157)
Supplement: Supplementary file 1 [file cimb-47-00157-s001.zip › cimb-3447794-supplementary.pdf]

S1. Supplementary Figure

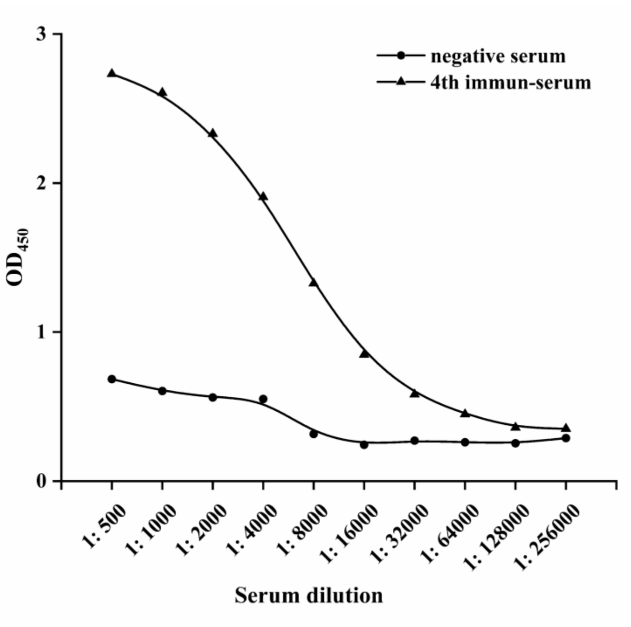

Figure S1. Serum titer of immunized alpaca

S2. National Center for Biotechnology Information database

XM_015252481.2 PREDICTED: Vicugna pacos immunoglobulin mu heavy chain (LOC102538064), partial mRNA

1 gaggagcccc agtccgggat tcccagctgc tcccattctc taaccaggac tgagcacaga
61 cgacccgcca tggagctggg gctgagtttg gtggtcctgg ctgctctttt acaaggtgtc
121 caggctgagg tgcagctcgt ggagtctggg ggaggcttgg tgcaggctgg ggggtctctg
181 agactctcct gtgcagcctc tggattcact ttcgatgatt atgccatagg ctggttccgc
241 caggccccag ggaaggagcg tgagggggtc tcatgtatta gtagtagtga tggtagcaca
301 tactatgcag actccgtgaa gggccgattc accatctcca gtgacaacgc caagaacacg
361 gtgtatctgc aaatgaacag cctgaaacct gaggacacgg ccgtttatta ctgtgcagca
421 gatctggcgg gcctcggctg tagtggtagt tactaccatg actactgggg ccaggggacc
481 caggtcaccg tctcctcaga gagctcgtct gccccgacac tcttccccct cgcctcctgt
541 gagagccccg tgtccgacga gagcccagtg gccttgggct gcctagcccg ggactccctg
601 cctggctcca tcaccttctc ctggagctac ccgaacggca tcgcggtcag tagccagagc
661 atcaagacct tcccgtccgt cctgcgggag ggcaagtatg tggccacctc ccaggtgctc
721 ctgccctccc agagcgtcct ccaggggtca gagctgattt gcaaagtcca gcactccaag
781 gggaactcgg acatggttgt gcccctccca gtgattttag atctgccccc cagcgtgaca
841 ctcttcatgc ccccccgaga tggcttctct ggcacttcca aacgcacgtc caagctcatc
901 tgtcaggcca cagacttcag ccccagggag atctccgtgt cctg
